# Evaluation of 34 Cytokines and Vitamin D Status Reveal A Sexually-Dimorphic Active Immune Response to SARS-CoV-2

**DOI:** 10.3390/healthcare10122571

**Published:** 2022-12-19

**Authors:** Osama E. Amer, Shaun Sabico, Eman Sheshah, Naif H Alotaibi, Dara A. Aldisi, Mushira A. Enani, Naji J. Aljohani, Naemah Alshingetti, Suliman Y. Alomar, Syed D. Hussain, Abdullah M. Alnaami, Mohamed A. Elsaid, Nasser M. Al-Daghri

**Affiliations:** 1 Biochemistry Department, College of Science, King Saud University, Riyadh 11451, Saudi Arabia; 2 Diabetes Care Center, King Salman Bin Abdul-Aziz Hospital, Riyadh 12769, Saudi Arabia; 3 Department of Medicine, College of Medicine, King Saud University, Riyadh 12372, Saudi Arabia; 4 Department of Community Health Sciences, College of Applied Medical Sciences, King Saud University, Riyadh 11451, Saudi Arabia; 5 Infectious Diseases Section, King Fahad Medical City, Riyadh 59046, Saudi Arabia; 6 Obesity, Endocrine and Metabolism Center, Department of Medicine, King Fahad Medical City, Riyadh 12231, Saudi Arabia; 7 Obstetrics and Gynecology Department, King Salman Bin Abdul-Aziz Hospital, Riyadh 11564, Saudi Arabia; 8 Department of Zoology, College of Science, King Saud University, Riyadh 11495, Saudi Arabia

**Keywords:** inflammation, severe acute respiratory syndrome, COVID-19, Arab, chemokines

## Abstract

Background: Several observational studies have inconsistently demonstrated that vitamin D deficiency is a risk factor for coronavirus disease-19 (COVID-19) infection and severity. Discrepancies in results may partially be explained by the individuals’ immune profiles, which are modulated, in varying degrees, by vitamin D status and sex hormones. Methods: In this study we evaluated the differences and associations of serum levels of 25(OH)D with 34 cytokines in 220 adults (82 controls (41 males; 41 females) and 138 SARS-CoV-2 patients (79 males and 59 females)) with and without COVID-19. Results: Serum 25(OH)D levels were significantly lower in the SARS-CoV-2 group than in the controls. Serum IP-10, MCP-1, CRP, IFNγ, IL-10, IL-13, IL-17α, IL-23, and IL-6 were significantly higher in COVID-19 patients compared to controls. Serum levels of VEGF, IFNγ, IL-13, and IL-5 were significantly higher in male patients than in females. 25(OH)D was significantly correlated with EFG (R = 0.39, p < 0.05) and IL-15 (R = 0.39, p < 0.05) in male patients, while it was inversely correlated with CRP (R = −0.51, p < 0.05) in female patients. Conclusions: Altered levels of cytokines, chemokines, and vitamin D were observed in SARS-CoV-2 adult patients. These expressions were sexually dimorphic and thus highlight the sex-specific nature of the active immune response following SARS-CoV-2 infection.

## 1. Introduction

The ongoing coronavirus disease 2019 (COVID-19) pandemic, now in its third year, has been responsible for an estimated 18 million human deaths, reported as excess mortality by the University of Washington [1]. The pathogen responsible for this global tragedy is the severe acute respiratory syndrome novel coronavirus 2 (SARS-CoV-2), the latest from a family of highly pathogenic human coronaviruses also including SARS-CoV and the Middle East respiratory syndrome coronavirus (MERS-CoV) [2]. COVID-19 can cause a wide range of clinical presentations, ranging from asymptomatic to severe viral pneumonia with respiratory failure and even death [3,4,5]. While there is abundant evidence on the clinical sequelae of COVID-19 infections, the pathogenesis of COVID-19 has not been well defined [6,7]. 

Previous data demonstrating associations between COVID-19 with cytokine and chemokine dysregulation are limited. However, clinical and in vitro studies suggest similarities between COVID-19 and previous global outbreaks (SARS-CoV and MERS-CoV) with regard to abnormally high levels of pro-inflammatory cytokines and chemokines [8]. In fact, hyperinflammation caused by cytokine release syndrome (CRS), as well as upregulation of inflammatory cytokines [4], are associated with the severity of COVID-19 disease [4,9]. Likewise, significant elevation of the pro-inflammatory cytokine profile has been reported as a key player in the severity and eventual death of patients with SARS-CoV [10], MERS-CoV [11], and SARS-CoV-2 diseases [7,10,11,12,13,14]. Furthermore, data from COVID-19 patients indicated high circulating levels of IL-10 and IL-4, which are protective against inflammation [15]. In SARS-CoV infection, elevated inflammatory cytokines cause a dysregulation of the systemic inflammatory response [15,16,17,18,19], leading to acute respiratory distress syndrome (ARDS), multiple organ failure, and even death. 

Vitamin D (25(OH)D) is a fat soluble secosteroid hormone which has both autocrine and endocrine functions through its vitamin D receptor (VDR), which is expressed in various cell types in the body [20,21,22]. Several studies reported that 25(OH)D has anti-inflammatory and immune-modulating properties which play a significant protective role in cardiovascular disease (CVD) among others [23,24]. Vitamin D supplementation has been demonstrated to have beneficial effects in increasing levels of anti-inflammatory markers and decreasing the production of inflammatory cytokines [25,26] in several studies. Moreover, 25(OH)D is generally accepted as an adjuvant management of COVID-19 as multiple studies showed an association between vitamin D status, susceptibility to viral infections, and COVID-19 severity [27,28,29]. 

We previously observed in several Arab cohorts that vitamin D status in SARS-CoV-2-positive participants was significantly lower than in their SARS-CoV-2 negative counterparts, but that this difference was not associated with COVID-19 susceptibility [27,28]. One reason that can potentially explain this lack of association may be differences in the individuals’ inflammatory and immune profiles. Another reason may be the individuals’ sex, since sex hormones largely impact immune response and strength of immunity [30]. As such, the present retrospective case–control study aims to compare variances in serum levels of cytokines and chemokines of adult male and female COVID-19 patients screened for SARS-CoV-2, and whether 25(OH)D levels are associated with these cytokines and chemokines.

## 2. Materials and Methods

### 2.1. Study Design and Participants

This multi-center case–control study included a total of 220 adult residents of Riyadh, Saudi Arabia, who were swabbed twice (nasopharyngeal and oropharyngeal) for SARS-CoV-2 (n = 138 reverse transcription-polymerase chain reaction (RT-PCR)-confirmed SARS-CoV-2-positive cases, and n = 82 SARS-CoV-2-negative cases as control group) at King Saud University Medical City-King Khalid University Hospital (KSUMC-KKUH) and King Salman Medical City (KSMC), Riyadh, Saudi Arabia, between May and July 2020. Confirmed COVID-19 cases were admitted for isolation and supportive care at a designated quarantine facility while RT-PCR-negative cases were discharged. No-one from the control group had had a prior COVID-19 infection. Children and pregnant women were excluded. Patients’ nasopharyngeal and oropharyngeal swab samples were obtained for RT-PCR analysis, which was performed following the manufacturer’s instructions by qualified laboratory personnel at the biosafety level 2-facility (BSL-2) in KSUMC-KKUH. Only asymptomatic to mild cases were included, which, according to the Ministry of Health, meant that the patients had no shortness of breath, required no oxygen on presentation, and showed no evidence of pneumonia, but presented with clinical symptoms such as fever [28,31]. Diagnosis of SARS-CoV-2 infection was based on national guidelines in Saudi Arabia [31]. Since the study was conducted during the first wave of the pandemic, COVID-19 vaccine status was not applicable.

All study participants provided hand-written informed consent prior to inclusion. A general questionnaire was administered, which included medical history (hypertension (HPN), diabetes mellitus (DM), congestive heart failure (CHF), cardiovascular disease (CVD), chronic kidney disease (CKD), and stroke, along with smoking history), as well as a list of medications (angiotensin receptor blockers (ARB), β-blockers, angiotensin converting enzyme (ACE) inhibitors, anti-coagulants, anti-platelet drugs, statins, calcium channel blockers (CCB), oral hypoglycemic agents, and insulin) prior to recruitment. Ethical approval was obtained from the Institutional Review Board (IRB) of the College of Medicine in KSUMC-KKUH in Riyadh, Saudi Arabia (E-20-4803, 11 June 2020).

### 2.2. Anthropometry and Blood Collection

Fasting and non-fasting blood samples were collected following nasal and oral swabs. Anthropometric measurements taken included height (cm), weight (kg), body mass index (BMI, kg/m2), and waist (cm) and hip (cm) circumferences. Trained nurses were assigned to take blood pressure (systolic and diastolic in mmHg) (mean of two readings) using standard procedures (Table S1).

### 2.3. Biochemical Analyses

Fasting glucose and lipid profile, including triglycerides, total cholesterol, LDL cholesterol (LDL-c), and HDL-c, were analyzed using a chemical analyzer (Konelab, Espoo, Finland). Total serum 25(OH)D was measured using commercial electrochemiluminescence immunoassay kits (Roche Diagnostics, Germany). The intra- and inter-assay coefficients of variation were 4.6% and 5.3%, respectively. Vitamin D deficiency (25(OH)D < 50 nmol/L) was defined based on national and regional recommendations [32,33]. A total of 34 serum cytokines, chemokines, and inflammatory proteins (EGF, FGF-2, TFGα, G-CSF, GMCSF, fractalkine, EOTAXIN, IFNα2, GRO, MCP-3, MDC, sCD40L, IL-15, IL-1ra, IL-1α, IL-1β, IL-2, IL-4, IL-7, CXCL-8, IP-10, MCP-1, MIP-1α, MIP-1β, VEGF, IFNγ, IL-10, IL-13, IL-17α, IL-23, IL-5, IL-6, and TNFα) were measured using multiplex assay kits (Milliplex^®^ human high-sensitivity T cell magnetic bead panel) that utilized the Luminex xMAP Technology platform (Luminex Corporation, TX, USA) according to the manufacturer’s instructions (Table 1). C-reactive protein (CRP) was measured using Maglumi CRP chemiluminescent immunoassays (CLIA) (Shenzhen New Industries Biomedical Engineering Co., Ltd. (SNIBE) Diagnostics, Shenzhen, China).

### 2.4. Data Analysis and Sample Size Determination

Data were analyzed using SPSS version 21. Results are presented as mean ± standard deviation for continuous normal variables and median (1st quartile–3rd quartile) for non-normal continuous variables. Independent sample T-test and Mann–Whitney U-test were used to examine SARS-CoV-2 and gender difference in continuous normal and non-normal biochemical variables, respectively. Analysis of covariance (ANCOVA) was used to obtain age and BMI adjustment. Logistic regression was used with forward stepwise selection with selected cytokines to identify the most significant associations with SARS-CoV-2. Pearson and Spearman correlation coefficients were used to test for association between vitamin D and other normal and non-normal biochemical parameters, respectively. Significance was set at *p* < 0.05.

A post-hoc power analysis revealed that this sample size achieves a power higher than 80% with an effect size of 0.196 (two-sided α of 0.05), as calculated using the G*Power.

## 3. Results

In relation to their function, the measured cytokines and chemokines were gathered into groups: Growth Factors (EGF, FGF-2, VEGF, and TFG-α), Colony Stimulating Factors (G-CSF and GM-CSF), Interleukins (IL-15, IL-1ra, IL-1α, IL-1β, IL-2, IL-4, IL-7, IL-10, IL-13, IL-17α, IL-23, IL-5, and IL-6), chemokines (IP-10, EOTAXIN, GRO, MCP-3, MDC, MCP-1, MIP-1α, MIP-1β, fractalkine, and CXCL-8), interferons (IFNγ and IFNα2), C-reactive protein (CRP), tumor necrosis factor (TNF-α), Soluble CD40 ligand (sCD40L).

### 3.1. Comparison of Serum Cytokine and Chemokine Levels in COVID-19 Patients and Controls

Table 2 shows clinical characteristics of 220 participants (82 controls and 138 COVID-19 patients). For the present study, serum cytokine and chemokine levels in COVID-19 patients were compared to those in controls. Serum levels of IFNγ, CRP, IL-10, IL-13, IL-6 (Figure S1A), IP10, MCP-1 and IL-23 (Figure S1B) were significantly higher in COVID-19 patients than in controls after adjustment for age and BMI. On the other hand, serum levels of EGF, TFGα, G-CSF, GM-CSF, fractalkine, EOTAXIN, IFNα2, MDC, sCD40L, IL-15, IL1ra, IL-1α, IL-1β, IL-2, IL-4, IL-7, CXCL-8, MIP-1α, and MIP-1β were significantly lower in COVID-19-positive patients compared to controls. There were no statistically significant differences in serum levels of FGF-2, GRO, MCP-3, VEGF, IL-5, and TNFα between both groups. Lastly, 25(OH)D levels were significantly lower in the COVID-19-positive group than in controls, after adjusting for age and BMI (adjusted *p* = 0.009). All other analyzed biochemical characteristics are shown in Table 2.

### 3.2. Comparison of Cytokine and Chemokine Expression According to SARS-CoV-2 Status and Sex

Table 3 shows age- and BMI-adjusted cytokine comparisons between cases and controls in males and females. Serum levels of EGF, G-CSF, GM-CSF, IL-1ra, IL-1α, IL-1β, IL-2, IL-4, IL-7, IL-8, EOTAXIN, fractalkine, GRO, P-10, MDC, MIP-1α, MIP-1β, IFNα2, and sCD40L were significantly lower in female COVID-19 patients compared to controls. Serum levels of IL-23 and CRP serum levels were significantly higher in female cases than controls. In male participants, EGF, GM-CSF, IL-1ra, IL-1α, IL-1β, IL-2, IL-4, IL-7, IL-15, EOTAXIN, MDC, MIP-1α, MIP-1β, IFNα2, and sCD40L were significantly lower in COVID-19 patients compared to controls. Furthermore, circulating IL-6, IL-10, IL-13, IL-23, IFNγ, and TNFα were significantly higher in male patients than in male controls. For all other measured biochemical parameters, results showed no significant differences.

### 3.3. Associations of Vitamin D Status, Cytokines, and Chemokines

No significant associations were observed between 25(OH)D and measured cytokines and chemokines regardless of SARS-CoV-2 status (Table S2). However, when stratified according to sex (Table 4), levels of 25(OH)D showed a significant positive correlation with EFG (R = 0.39, *p* < 0.05) and IL-15 (R = 0.39, *p* < 0.05), and a borderline significant association with IFNγ (R = 0.33, *p* = 0.06) in male patients with COVID-19. In female patients with COVID-19, 25(OH)D was inversely correlated with CRP (R = −0.51, *p* < 0.05), and borderline significant inverse correlations with IL-2 (R = −0.5, *p* = 0.06) and IL-17α (R = −0.36, *p* = 0.06) were observed. For males without COVID-19, 25(OH)D was inversely associated with GRO (R = −0.60, *p* < 0.01) and positively associated with VEGF (R = 0.62, *p* < 0.01). In females without COVID-19, 25(OH)D had a significant positive correlation with IP-10 (R = 0.36, *p* < 0.05).

Logistic regression was used with forward stepwise selection to identify the cytokines with the highest association with SARS-CoV-2 (Table 5). All cytokines that were significantly elevated in SARS-CoV-2 patients were entered into the regression along with age and BMI. Results showed that only IP-10, IFNγ and IL-23 were independently associated with SAR-CoV-2. IFNγ was the cytokine showing the highest association with COVID-19 infection, and was therefore considered a significant risk factor for COVID-19 in our cohort (OR 169.2 (95% CI 5.5–5200.4); *p* = 0.003)).

## 4. Discussion

The present study correlated differences in serum levels of 25(OH)D with 34 cytokines and chemokines in a cohort of 220 adult Arab males and females (138 COVID-19 patients and 82 controls) in Riyadh, Saudi Arabia. Studies from previous epidemics revealed that inflammatory responses and abnormal cytokine profiles were correlated with acute lung injury and pulmonary inflammation in SARS- [10] and MERS-CoV [11] infections. In the present study, we detected significantly higher serum levels of measured cytokines, chemokines, and inflammatory proteins (IP-10, CRP, IFNγ, IL-10, IL-13, IL-17α, IL-23, and IL-6) in COVID-19 patients than in controls. SARS-CoV-2 infection induces over-production of pro-inflammatory cytokines and chemokines as a defense mechanism against the viral infection. On the other hand, a dysregulation, i.e., hyper-inflammation, results in cytokine release syndrome (CRS) leading to apoptosis of immune cells, impaired cytotoxic function, and tissue damage [13]. More importantly, our results showed that male patients had significantly higher serum concentrations of VEGF, IFNγ, IL-13, and IL-5 than female patients. This is in line with previous studies demonstrating a stronger response in cytokines and chemokines in SARS-CoV-2-infected male than in female patients [6,13]. Differences in the expression of cytokines and chemokines secondary to infection are partially yet substantially controlled by sex hormones, which modulate the innate immune function at various stages of human life concurrent with hormonal changes. This can explain why males and females may present different clinical symptoms and time-to-recovery given the same infection [6,34,35,36]. 

Our data indicated an elevation in IP-10/CXCL-10, CRP, IL-10, and IL-6. A study by Notz et al. on 39 patients admitted to the intensive care unit with confirmed COVID-19, reported that IL-6, CRP, and IL-10 levels were substantially increased in patients in comparison to controls [37]. IP-10 is produced by monocytes and macrophages and has been suggested as a biomarker for severity in COVID-19 [38]. Previous studies reported that anti-retrovirus treatment reduced plasma IP-10 indicating an important role of IP-10 in the pathogenesis of the diseases [38]. Huang et al. [7] showed a significant increase in IFN-γ, IP-10, and MCP-1 levels in COVID-19 patients.

Remarkably, our results showed decreased serum levels of TNFα, IL-4, IL-2, IL-1β, IL-2, IL-7, and GM-CSF in COVID-19 patients compared to controls, which indicates that the cytokine profile in mild cases of COVID-19 does not match a classical cytokine storm where TNFα is considered the leading cytokine and a therapeutic target. Recently, Liu et al. detected that levels of TNFα and IFNγ did not increase in mild COVID-19 [39]. However, to maintain balance between pro- and anti-inflammatory mechanisms is a significant factor in a robust immune response. Cytokines and chemokines are vital to initiate and augment the response of the innate and adaptive immune system [40]; concomitant hyper-inflammation has been linked to severe pulmonary dysfunction though [41].

25(OH)D serum levels were significantly lower in COVID-19 patients than in controls after adjusting for the main confounding factors. In addition, 25(OH)D was positively correlated with EFG and IL-15 in male patients, and inversely correlated with CRP in female patients. In a retrospective study on 107 individuals screened for COVID-19 (27 RT-PCR positive and 80 negative), the authors reported that COVID-19 patients had lower median 25(OH)D levels (11.1 ng/mL) compared to their RT-PCR-negative counterparts (24.6 ng/mL) [42]. Recently, Ilie et al. detected that serum 25(OH)D levels were significantly associated with COVID-19 number of cases and mortality in some European countries [43]. Additionally, experimental studies showed that 25(OH)D can inhibit the inflammatory cytokines IL-6 and TNFα by attenuating the activation of p38 MAP kinase in human macrophages/monocytes. Moreover, 25(OH)D promotes the stimulation of T regulatory cells, thus inhibiting production of pro-inflammatory cytokines, including IL-21, IFNγ, and IL-17 [44]. However, recently, Hastie et al. reported no relationship between 25(OH)D serum levels and COVID-19 risk in a cohort of more than 340,000 participants from the UK Biobank [45]. Despite the inconsistencies in associations, the use of vitamin D as an adjuvant therapy for COVID-19 has merits, as it has been observed to resolve COVID-19 symptoms faster even in mild–moderate cases known to have vitamin D deficiency [46,47].

The authors acknowledge several limitations that should be considered in interpreting the findings. Corrections for multiple comparisons were not performed given the small sample size. However, we believe this approach only increases the likelihood of type 2 errors and truly significant differences become non-significant [48]. Another limitation is that the actual virus loads were not obtained since all SARS-Cov-2 testing was centralized by the Ministry of Health, hence correlations with viral infection/replication were not performed and analysis was limited to only presence and absence of SARS-CoV-2. Nevertheless, the present study is one of the few to demonstrate differences in the circulating cytokine and chemokine profiles of male and female Arab adult patients with and without COVID-19 and their associations with serum 25(OH)D levels. A prospective design with a larger cohort could yield temporal changes in the immune response after infection with COVID-19.

## 5. Conclusions

Our results indicated a sexual dimorphism in the expression of circulating 25(OH)D, cytokines, and chemokines among COVID-19 patients as compared to controls, suggesting a sex-bias in the active immune response. Serum levels of IP-10, CRP, IL-10, IFNγ, IL-13, IL-17α, IL-23, IL-6 were particularly upregulated in COVID-19 patients compared to controls. Further studies are needed to prospectively investigate and consider the role of sexual dimorphism in the strength of innate and adaptive immunity as it may play a pivotal role in vaccine efficiency and overall immune response.

## Figures and Tables

**Table 1:** List of 34 cytokines assessed with intra- * and inter-assay ** CV.

#	Parameters		*	**
Growth Factors		Definition		
1	EGF (pg/mL)	Epidermal growth factor	2.3	5.8
2	FGF-2 (pg/mL)	Basic fibroblast growth factor 2	2.3	4.8
3	TGFα (pg/mL)	Transforming growth factor alpha	4.1	9.5
4	VEGF (pg/mL)	Vascular endothelial growth factor	3.7	10.4
Colony Stimulating Factors				
5	G-CSF (pg/mL)	Granulocyte colony-stimulating factor	1.8	15.5
6	GM-CSF (pg/mL)	Granulocyte macrophage colony-stimulating factor	3.1	10.1
Interleukins				
7	IL-1ra (pg/mL)	Interleukin-1 receptor antagonist	2.1	10.7
8	IL-1α (pg/mL)	Interleukin-1 alpha	3.3	12.8
9	IL-1β (pg/mL)	Interleukin-1 beta	2.3	6.7
10	IL-2 (pg/mL)	Interleukin-2	2.1	6.3
11	IL-4 (pg/mL)	Interleukin-4	2.9	14.2
12	IL-5 (pg/mL)	Interleukin-5	2.6	10.8
13	IL-6 (pg/mL)	Interleukin-6	2.0	18.3
14	IL-7 (pg/mL)	Interleukin-7	1.7	16.1
15	IL-8 (pg/mL)	Interleukin-8	1.9	3.5
16	IL-10 (pg/mL)	Interleukin-10	1.6	16.8
17	IL-13 (pg/mL)	Interleukin-13	2.2	9.2
18	IL-15 (pg/mL)	Interleukin-15	2.7	8.1
19	IL-17α (pg/mL)	Interleukin-17 alpha	2.2	7.9
20	IL-23 (pg/mL)	Interleukin-23	3.2	5.1
Chemokines				
21	EOTAXIN (pg/mL)		7.2	10.8
22	Fractalkine (pg/mL)		4.5	9.4
23	GRO (pg/mL)	Growth-regulated oncogene	2.1	9.2
24	IP-10 (pg/mL)	Interferon-inducible protein 10	2.6	15.3
25	MCP-3 (pg/mL)	Monocyte chemotactic protein-3	1.6	6.4
26	MDC (pg/mL)	Macrophage-derived chemokine	1.6	7.2
27	MCP-1 (pg/mL)	Monocyte chemoattractant protein-1	1.5	7.9
28	MIP-1α (pg/mL)	Macrophage inflammatory protein-1 alpha	1.9	14.5
29	MIP-1β (pg/mL)	Macrophage inflammatory protein-1 beta	2.4	8.8
Interferons				
30	IFNγ (pg/mL)	Interferon gamma	1.6	12.0
31	IFNα2 (pg/mL)	Interferon alpha 2	2.4	13.3
Inflammatory Cytokines				
32	sCD40L (pg/mL)	Soluble CD40 Ligand	3.7	18.9
Inflammatory Proteins				
33	TNFα (pg/mL)	Tumor necrosis factor alpha	2.6	13.0
34	CRP (ug/dL)	C-reactive protein	4.4	6.6

* intra-assay CV (%); ** inter-assay CV (%).

**Table 2:** Biochemical characteristics of study participants according to their SARS-CoV-2 status.

Parameters	Overall	SARS-CoV-2 Status		*p*-Value	*p*-Value *
		Negative	Positive		
N	220	82 (37.3)	138 (62.7)		
Age (years)	43.2 ± 15.3	32.3 ± 13.1	49.7 ± 12.6	<0.001	--
BMI (kg/m^2^)	28.1 ± 5.5	26.6 ± 5.1	28.9 ± 5.5	<0.001	--
WHR	1.0 ± 0.1	1.0 ± 0.1	1.0 ± 0.1	0.45	0.13
Systolic BP (mmHg)	126.0 ± 16.2	119.0 ± 9.6	130.1 ± 17.8	<0.001	<0.001
Diastolic BP (mmHg)	75.4 ± 11.2	75.7 ± 8.7	75.2 ± 12.4	0.75	0.75
Temperature (°C)	37.1 ± 1.1	36.6 ± 0.5	37.5 ± 1.2	<0.001	<0.001
Pulse rate	92.5 ± 16.4	94.0 ± 15.3	91.6 ± 16.9	0.31	0.77
Respiratory rate	22.3 ± 3.9	19.7 ± 2.6	23.5 ± 3.8	<0.001	<0.001
25(OH)D (nmol/L)	57.5 ± 27.5	61.8 ± 22.8	55.0 ± 28.8	0.06	0.009
Growth factors					
EGF (ng/mL)	0.8 (0.4–1.2)	0.9 (0.5–1.2)	0.6 (0.3–1.2)	0.004	0.001
FGF-2 (ng/mL)	0.2 (0.2–0.3)	0.3 (0.2–0.4)	0.2 (0.2–0.3)	0.001	0.16
TGFα (pg/mL)	13.3 (6–24)	18.7 (8–25)	10.4 (6–16)	0.006	<0.001
VEGF (ng/mL)	0.5 (0.2–0.9)	0.5 (0.2–0.9)	0.5 (0.3–1.0)	0.25	0.94
Colony Stimulating Factors					
G-CSF (pg/mL)	89.8 (43–178)	129.6 (75–194)	55.2 (29–122)	<0.001	0.006
GM-CSF (pg/mL)	25.7 (12–43)	34.2 (21–58)	14.4 (8–24)	<0.001	<0.001
Interleukins					
IL-1ra (pg/mL)	145.9 (66–280)	200.9 (125–401)	86.0 (44–140)	<0.001	<0.001
IL-1α(pg/mL)	130.7 (56–252)	233.4 (134–435)	48.5 (10–80)	<0.001	<0.001
IL-1β (pg/mL)	3.0 (1–8)	7.7 (4–10)	1.1 (0.4–2)	<0.001	<0.001
IL-2 (pg/mL)	7.7 (2–25)	16.8 (9–29)	2.0 (0.7–4)	<0.001	<0.001
IL-4 (pg/mL)	224 (112–456)	387.6 (264–548)	98.1 (52–141)	<0.001	<0.001
IL-5 (pg/mL)	2.8 (2–5)	3.2 (2–5)	2.5 (1.8–4)	0.14	0.36
IL-6 (pg/mL)	5.2 (2–13)	3.1 (2–7)	10.0 (5–20)	<0.001	0.05
IL-7 (pg/mL)	33.2 (18–66)	52.2 (30–88)	16.7 (9–28)	<0.001	<0.001
IL-8 (pg/mL)	26.6 (17–49)	28.6 (20–40)	21.7 (14–56)	0.07	0.01
IL-10 (pg/mL)	13.7 (8–24)	9.9 (6–17)	21.7 (9–32)	<0.001	0.01
IL-13 (pg/mL)	5.6 (3–10)	4.2 (2–6)	9.6 (6–18)	<0.001	<0.001
IL-15 (pg/mL)	13.7 (8–30)	22.1 (11–36)	10.9 (5–17)	<0.001	0.001
IL-17α (pg/mL)	5.8 (4–8)	5.8 (4–8)	6.0 (4–8)	<0.001	0.72
IL-23 (pg/mL)	187.1 (93–276)	129.5 (72–235)	236.3 (170–310)	<0.001	<0.001
Chemokines					
EOTAXIN (pg/mL)	96.9 (58–185)	124.8 (76–249)	60.5 (28–120)	<0.001	<0.001
Fractalkine (pg/mL)	333.2 (157–564)	454.1 (216–797)	208.6 (145–345)	<0.001	0.003
GRO (ng/mL)	4.9 (3.9–6.8)	5.1 (4.2–6.9)	4.7 (3.5–5.8)	0.34	0.49
IP-10 (ng/mL)	0.4 (0.2–0.5)	0.4 (0.3–0.6)	0.2 (0.1–0.4)	0.18	0.004
MCP-3 (pg/mL)	36.3 (19–55)	42.2 (22–65)	26.7 (15–40)	0.07	0.22
MDC (ng/mL)	1.8 (0.9–2.7)	2.6 (1.8–3.2)	0.9 (0.4–1.4)	<0.001	<0.001
MCP-1 (ng/mL)	0.9 (0.7–1.6)	1.0 (0.8–1.6)	0.8 (0.5–1.4)	0.06	0.03
MIP-1α (pg/mL)	17.7 (9.9–25)	22.1 (17–29)	10.4 (6–18)	<0.001	<0.001
MIP-1β (pg/mL)	54.9 (36–82)	67.6 (45–94)	42.3 (32–66)	<0.001	<0.001
Interferons					
IFNγ (pg/mL)	8.0 (5–15)	6.1 (4–9)	17.0 (6–25)	<0.001	<0.001
IFNα2 (pg/mL)	70.0 (28–145)	100.0 (58–197)	26.1 (16–46)	<0.001	<0.001
Inflammatory Cytokines					
sCD40L (pg/mL)	58.3 (16–371)	184.6 (61–975)	14.3 (10–34)	<0.001	<0.001
TNFα (pg/mL)	7.9 (6–12)	7.2 (5–10)	9.5 (7–16)	0.07	0.24
Inflammatory Proteins					
CRP (µg/mL)	10.2 (0.9–32)	2.3 (0.8–14)	42.0 (6–75)	<0.001	0.002

Data are presented as mean ± SD for normal variables, and median (1st quartile–3rd quartile) for non-normal variables, * indicates *p*-value adjusted for age and BMI. *p* < 0.05 is considered significant.

**Table 3:** Clinical differences in male and female participants.

Parameters	Females				Males			
	Control	Case	*p*-Value	*p*-Value *	Control	Case	*p*-Value	*p*-Value *
N	41	59			41	79		
Age (years)	33.2 ± 12.3	49.1 ± 12.4	<0.001	--	31.4 ± 13.9	50.1 ± 12.8	<0.001	--
BMI (kg/m^2^)	26.4 ± 4.8	29.9 ± 6.4	0.003	--	26.9 ± 5.4	28.2 ± 4.6	0.17	--
WHR	1.0 ± 0.1	1.0 ± 0.1	0.70	0.13	1.0 ± 0.1	1.0 ± 0.1	0.39	0.50
Systolic BP (mmHg)	116.5 ± 10.3	128.9 ± 19.3	<0.001	0.15	121.4 ± 8.4	130.9 ± 16.7	0.001	0.22
Diastolic BP (mmHg)	74.0 ± 9.1	73.7 ± 13.5	0.92	0.63	77.5 ± 7.9	76.3 ± 11.6	0.55	0.40
Temperature (°C)	36.6 ± 0.5	37.3 ± 0.8	<0.001	<0.001	36.7 ± 0.6	37.6 ± 1.4	<0.001	0.001
Pulse rate	96.2 ± 9.0	90.4 ± 15.5	0.02	0.23	91.5 ± 20.2	92.4 ± 17.9	0.80	0.22
Respiratory rate	20.0 ± 0.2	22.7 ± 3.3	<0.001	<0.001	19.3 ± 3.9	24.0 ± 4.1	0.81	<0.001
25(OH) D (nmol/L)	65.0 ± 26.9	58.5 ± 19.8	0.36	0.03	59.0 ± 25.9	51.5 ± 17.5	0.09	0.21
Growth factors								
EGF (ng/mL)	0.9 (0.5–1.2)	0.4 (0.2–1.1)	0.003	<0.001	0.8 (0.5–1.1)	0.8 (0.3–1.5)	0.27	0.02
FGF-2 (ng/mL)	0.3 (0.2–0.4)	0.2 (0.2–0.3)	0.04	0.35	0.3 (0.2–0.3)	0.2 (0.2–0.2)	0.02	0.40
TGFα (pg/mL)	22.4 (11–30)	10.7 (7–15)	0.09	0.09	15.4 (7–24)	6.7 (6–17)	0.04	0.21
VEGF (ng/mL)	0.4 (0.3–0.7)	0.3 (0.2–0.7)	0.62	0.33	0.5 (0.2–0.9)	0.7 (0.4–1.1)	0.07	0.50
Colony Stimulating factors								
G-CSF (pg/mL)	151.7 (92–194)	61.3 (31–122)	0.003	0.05	88.5 (54–183)	54.2 (25–151)	0.04	0.06
GM-CSF (pg/mL)	40.1 (22–77)	16.6 (11–24)	0.001	0.05	32.1 (21–44)	11.6 (7–25)	<0.001	0.001
Interleukins								
IL-1ra (pg/mL)	289.6 (187–559)	125.9 (76–176)	0.001	0.03	176.9 (110–268)	64.0 (34–128)	0.001	0.004
IL-1α (pg/mL)	191.4 (134–440)	68.8 (41–83)	<0.001	<0.001	240.5 (137–385)	33.8 (7–76)	<0.001	0.001
IL-1β (pg/mL)	7.7 (3–12)	1.2 (0.7–2)	<0.001	0.011	6.7 (4–10)	0.8 (0.3–1.5)	<0.001	<0.001
IL-2 (pg/mL)	25.4 (15–32)	2.6 (1.5–4.0)	<0.001	<0.001	12.2 (6–22)	1.4 (0.6–2.5)	<0.001	<0.001
IL-4 (pg/mL)	383.1 (272–550)	100.6 (66–143)	<0.001	<0.001	388.5 (249–519)	93.7 (51–135)	<0.001	<0.001
IL-5 (pg/mL)	3.3 (2–5)	2.2 (1–3)	0.04	0.09	3.2 (2–5)	3.2 (2–5)	0.96	0.57
IL-6 (pg/mL)	3.2 (2.5–6.5)	8.2 (3–16)	0.06	0.83	3.0 (2–9)	13.7 (8–22)	<0.001	0.01
IL-7 (pg/mL)	63.9 (34–112)	15.0 (7–26)	<0.001	<0.001	49.9 (26–73)	17.6 (9–30)	<0.001	<0.001
IL-8 (pg/mL)	34.5 (20–56)	21.3 (11–57)	0.12	0.046	25.7 (18–36)	21.8 (16–49)	0.40	0.20
IL-10 (pg/mL)	13.2 (6–20)	13.9 (8.1–30)	0.34	0.83	9.8 (5–15)	24.0 (14–32)	<0.001	0.001
IL-13 (pg/mL)	4.2 (2–6)	6.1 (3–15)	0.06	0.47	4.1 (3–6)	14.1 (8–20)	<0.001	<0.001
IL-15 (pg/mL)	22.1 (11–56)	15.5 (10–31)	0.10	0.25	21.6 (11–35)	8.1 (3.7–14)	0.002	0.003
IL-17α (pg/mL)	6.2 (4–8)	5.2 (4–7)	0.99	0.96	5.5 (3–8)	6.9 (5–10)	0.06	0.58
IL-23 (pg/mL)	132.2 (72–235)	198.5 (134–294)	0.02	0.030	124.2 (78–222)	250.9 (198–315)	0.002	0.003
Chemokines								
EOTAXIN (pg/mL)	135.1 (83–268)	57.8 (31–99)	<0.001	<0.001	105.6 (75–202)	68.3 (27–175)	0.010	0.001
Fractalkine (pg/mL)	483.9 (333–888)	201.9 (123–345)	<0.001	0.002	353.1 (139–743)	215.3 (145–346)	0.18	0.22
GRO (ng/mL)	5.4 (4.4–6.9)	4.9 (3.5–5.8)	0.04	0.005	4.6 (3.7–6.7)	4.7 (3.8–5.8)	0.87	0.34
IP-10 (ng/mL)	0.4 (0.2–0.6)	0.2 (0.1–0.3)	0.27	0.02	0.5 (0.3–0.6)	0.3 (0.2–0.5)	0.38	0.09
MCP-3 (pg/mL)	53.4 (25–77)	20.5 (15–38)	0.008	0.12	34.7 (15–55)	32.8 (14–41)	0.83	0.98
MDC (ng/mL)	2.6 (1.7–3.1)	1.2 (0.5–1.5)	<0.001	<0.001	2.6 (1.8–3.7)	0.7 (0.4–1.1)	<0.001	<0.001
MCP-1 (ng/mL)	1.0 (0.8–1.6)	1.2 (0.6–1.5)	0.33	0.30	1.0 (0.7–1.7)	0.7 (0.5–1.2)	0.12	0.05
MIP-1α (pg/mL)	24.2 (18–32)	13.9 (7–24)	0.01	0.05	20.3 (15–27)	9.6 (6–15)	<0.001	<0.001
MIP-1β (pg/mL)	75.6 (47–115)	42.7 (34–63)	0.002	<0.001	54.7 (42–77)	41.4 (32–77)	0.06	0.04
Interferons								
IFNγ (pg/mL)	6.1 (4–10)	12.4 (5–20)	0.002	0.05	6.1 (4–8)	21.0 (9–31)	<0.001	<0.001
IFNα2 (pg/mL)	124.4 (46–252)	32.4 (25–69)	0.001	0.028	81.1 (58–195)	19.7 (15–30)	<0.001	0.003
Inflammatory Cytokines								
sCD40L (pg/mL)	184.6 (99–975)	18.1 (10–43)	<0.001	<0.001	222.8 (47–1235)	13.8 (10–23)	<0.001	<0.001
TNFα (pg/mL)	7.9 (6–11)	8.8 (6–15)	0.627	0.74	6.9 (5–9)	12.3 (8–16)	<0.001	0.002
Inflammatory Proteins								
CRP (µg/mL)	2.2 (0.9–12.5)	66.4 (4.6–81.6)	<0.001	0.013	4.1 (0.7–14.7)	31.1 (6.2–63.7)	<0.001	0.053

Note: data are presented as mean ± SD for normal variables and median (1st Quartile–3rd Quartile) for non-normal variables. *p*-value < 0.05 is considered significant, * indicates adjusted for age and BMI.

**Table 4:** Correlation between Vitamin D and selected parameters according to COVID-19 and gender status.

Parameters	Control				Case			
	Female		Male		Female		Male	
	R	*p*-Value	R	*p*-Value	R	*p*-Value	R	*p*-Value
Age (years)	0.48 **	0.00	−0.13	0.44	0.09	0.54	0.19	0.12
BMI (kg/m^2^)	0.15	0.37	−0.04	0.83	0.03	0.82	0.01	0.94
WHR	0.18	0.30	0.02	0.92	0.03	0.89	0.09	0.60
Systolic BP (mmHg)	0.14	0.41	0.04	0.82	0.03	0.84	0.20	0.10
Diastolic BP (mmHg)	−0.06	0.74	0.24	0.15	−0.02	0.92	−0.09	0.46
Temperature (°C)	0.11	0.52	0.07	0.71	0.07	0.65	−0.03	0.81
Pulse rate	0.20	0.22	0.29	0.11	0.13	0.36	−0.07	0.57
Respiratory rate	−0.01	0.96	−0.10	0.64	0.55 **	0.00	0.19	0.12
Growth factors								
EGF (ng/mL)	−0.19	0.25	−0.12	0.49	−0.30	0.19	0.39 *	0.05
F-GF2 (pg/mL)	−0.29	0.08	0.05	0.78	0.28	0.31	−0.05	0.81
TFGα (ng/mL)	−0.04	0.80	0.01	0.96	0.09	0.71	0.20	0.30
VEGF (ng/mL)	−0.10	0.58	0.62 **	0.00	0.15	0.52	0.11	0.64
Colony Stimulating Factors								
G-CSF (pg/mL)	−0.09	0.64	0.13	0.52	−0.01	0.96	−0.34	0.14
GM-CSF (pg/mL)	−0.17	0.32	−0.09	0.62	0.14	0.65	−0.13	0.55
Interleukins								
IL-1ra (pg/mL)	−0.16	0.46	0.08	0.73	0.15	0.62	0.01	0.97
IL-1α (pg/mL)	−0.05	0.84	0.25	0.43	0.33	0.42	0.50	0.07
IL-1β (pg/mL)	−0.07	0.75	0.08	0.74	−0.33	0.23	−0.02	0.94
IL-2 (pg/mL)	−0.13	0.49	0.10	0.59	−0.50	0.06	0.08	0.75
IL-4 (pg/mL)	−0.19	0.26	−0.12	0.48	0.05	0.84	0.34	0.09
IL-5 (pg/mL)	−0.06	0.73	0.14	0.42	0.05	0.82	0.15	0.46
IL-6 (pg/mL)	0.00	0.99	0.14	0.42	−0.23	0.27	0.14	0.49
IL-7 (pg/mL)	−0.16	0.33	−0.10	0.61	0.20	0.41	0.19	0.39
IL-8 (pg/mL)	0.21	0.20	−0.19	0.27	0.06	0.80	0.13	0.52
IL-10 (pg/mL)	0.07	0.68	0.00	1.00	0.19	0.39	0.22	0.26
IL-13 (pg/mL)	0.17	0.29	0.07	0.71	−0.07	0.73	0.19	0.34
IL-15 (pg/mL)	0.02	0.91	0.30	0.20	−0.23	0.37	0.39 *	0.04
IL-17α (pg/mL)	−0.05	0.75	−0.25	0.14	−0.36	0.06	0.10	0.63
IL-23 (pg/mL)	−0.08	0.63	0.01	0.94	0.03	0.87	0.19	0.33
Chemokines								
EOTAXIN (pg/mL)	−0.20	0.22	−0.04	0.80	0.05	0.84	0.06	0.78
Fractalkine (pg/mL)	−0.18	0.31	0.14	0.48	0.01	0.96	−0.08	0.75
GRO (ng/mL)	0.08	0.66	−0.60 **	0.00	0.39	0.15	0.16	0.54
IP-10 (ng/mL)	0.36 *	0.03	−0.05	0.79	−0.10	0.69	0.04	0.84
MCP-3 (pg/mL)	−0.32	0.09	−0.06	0.79	−0.05	0.89	0.23	0.46
MDC (ng/mL)	−0.16	0.35	−0.11	0.53	0.13	0.59	−0.27	0.18
MCP-1 (ng/mL)	0.01	0.97	0.22	0.20	−0.19	0.42	−0.25	0.22
MIP-1α (pg/mL)	−0.21	0.24	0.12	0.52	−0.29	0.26	0.37	0.07
MIP-1β (pg/mL)	−0.10	0.54	0.13	0.46	−0.11	0.67	−0.14	0.51
Interferons								
IFNγ (pg/mL)	0.07	0.69	−0.18	0.31	−0.11	0.61	0.33	0.06
IFNα2 (pg/mL)	−0.23	0.20	0.08	0.67	−0.20	0.52	−0.24	0.33
Inflammatory Cytokines								
sCD40L (pg/mL)	−0.01	0.95	0.06	0.72	−0.34	0.14	−0.17	0.40
TNFα (pg/mL)	−0.01	0.94	0.18	0.28	−0.16	0.45	0.16	0.40
Inflammatory Proteins								
CRP (ug/mL)	0.14	0.41	0.19	0.27	−0.51*	0.04	0.12	0.57

Note: Data are presented as Spearman correlations. * *p* < 0.01, ** *p* < 0.05

**Table 5:** Associations between COVID-19 status and cytokines.

Parameters	OR (95% CI)	*p*-Value
Age (years)	1.1 (1.0–1.1)	0.007
BMI (kg/m^2^)	1.0 (0.9–1.2)	0.51
Log IP-10 (ng/mL)	0.1 (0.0–0.3)	0.002
Log IFNγ (ng/mL)	169.2 (5.5–5200.4)	0.003
Log IL-23 (ng/mL)	15.5 (1.0–250.7)	0.05

Note: Data are presented as OR (95%CI) obtained from logistic regression. *p* < 0.05 is considered significant.

## Data Availability

The data presented in this study are available on request from the corresponding author. The data are not publicly available due to privacy protection.

## Supplementary Materials

The following supporting information can be downloaded at: https://www.mdpi.com/article/10.3390/healthcare10122571/s1, Figure S1: Serum cytokine and chemokine levels of SARS-CoV-2 patients (positive) and healthy controls (negative); Table S1: General anthropometric and clinical characteristics of study participants; Table S2: Correlation between vitamin D and selected study parameters according to COVID-19 status.
