# Supplementary material for: Evaluation of 34 Cytokines and Vitamin D Status Reveal A Sexually-Dimorphic Active Immune Response to SARS-CoV-2
Source: Healthcare (Basel). 2022 Dec 19;10(12):2571. doi: 10.3390/healthcare10122571 (PMC9778313; doi:10.3390/healthcare10122571)
Supplement: Supplementary file 1 [file healthcare-10-02571-s001.zip › healthcare-2115081-supplementary.pdf]

Supplementary Table S1. General anthropometric and clinical characteristics of study participants

Parameters	Negative	Positive	P-value
**N**	**82 (37.3)**	**138 (62.7)**	
**Demographics**			
Male/Female	41/41	79/59	0.30
Saudi	57 (69.5)	82 (59.4)	0.13
Non-Saudi	25 (30.5)	56 (40.6)	
Age (years)	32.3 ± 13.1	49.7 ± 12.6	<0.001
BMI (kg/m^2)	26.6 ± 5.1	28.9 ± 5.5	<0.001
WHR	1.0 ± 0.1	1.0 ± 0.1	0.45
Systolic BP (mmHg)	119.0 ± 9.6	130.1 ± 17.8	<0.001
Diastolic BP (mmHg)	75.7 ± 8.7	75.2 ± 12.4	0.75
**Vital Signs**			
Temperature (^{0}C)	36.6 ± 0.5	37.5 ± 1.2	<0.001
Pulse Rate	94.0 ± 15.3	91.6 ± 16.9	0.31
Respiratory Rate	19.7 ± 2.6	23.5 ± 3.8	<0.001
**Medical History of Patients**			
Obesity	21 (27.6)	48 (36.4)	0.20
DM	14 (17.7)	88 (68.2)	<0.001
Hypertension	5 (6.1)	48 (34.8)	<0.001
Cancer	4 (4.9)	0 (0.0)	0.06
RA	2 (2.4)	3 (2.2)	0.90
CLD	0 (0.0)	1 (0.7)	0.72
Thyroid Disease	2 (2.4)	3 (2.2)	0.90
Epilepsy	0 (0.0)	2 (1.4)	0.48
Chronic Immobilization	0 (0.0)	1 (0.7)	0.72
Asthma	1 (1.2)	9 (6.5)	0.10
Hyperlipidemia	0 (0.0)	14 (10.1)	0.04
Heart Disease	0 (0.0)	10 (7.2)	0.07
Anemia	3 (3.7)	0 (0.0)	0.10
CKD	0 (0.0)	5 (3.6)	0.19
Hypertriglyceridemia	18 (22.8)	48 (36.9)	0.04
Low HDL	28 (35.4)	106 (81.5)	<0.001
**Supplementation**			
Using Vitamin C before Pandemic	1 (1.2)	0	0.32
Using Vitamin C after Pandemic	6 (7.3)	79 (57.2)	<0.001
Using Vitamin D before Pandemic	3 (3.7)	0	0.08
Using Vitamin D after Pandemic	1 (1.2)	57 (41.3)	<0.001

Note: Data presented as N (%). p<0.05 considered significant.

Supplementary Table S2. Correlation between vitamin D and select study parameters according to COVID-19 status

Parameters	Control		Case	
	R	P	R	P
Age (years)	0.17	0.15	0.15	0.11
BMI (kg/m^2)	0.04	0.72	0.03	0.73
WHR	0.18	0.13	0.06	0.65
Systolic BP (mmHg)	0.15	0.22	0.10	0.27
Diastolic BP (mmHg)	0.08	0.51	-0.04	0.67
Temperature (‘C)	0.08	0.51	0.00	0.97
Pulse Rate	0.26*	0.032	-0.01	0.94
Respiratory Rate	0.01	0.96	0.32**	0.000
**Cytokines**				
EGF (pg/ml)	-0.14	0.25	0.05	0.74
FGF2 (pg/ml)	-0.21	0.07	0.07	0.68
EOTAXIN (pg/ml)	-0.14	0.25	0.05	0.76
TFGα (pg/ml)	-0.02	0.83	0.14	0.33
GCSF (pg/ml)	-0.07	0.62	-0.18	0.28
GMCSF	-0.16	0.18	0.01	0.96
Fractalkine (pg/ml)	-0.07	0.61	-0.02	0.91
IFNα2 (pg/ml)	-0.17	0.16	-0.15	0.41
GRO (pg/ml)	-0.19	0.15	0.27	0.12
MCP3 (pg/ml)	-0.27*	0.049	0.00	1.00
MDC (pg/ml)	-0.14	0.24	-0.13	0.39
IL15 (pg/ml)	0.07	0.63	0.16	0.28
sCD40L (pg/ml)	0.00	0.98	-0.25	0.09
IL1RA (pg/ml)	-0.16	0.28	0.05	0.76
IL1a (pg/ml)	0.05	0.78	0.35	0.12
IL1b (pg/ml)	-0.05	0.77	-0.21	0.26
IL2 (pg/ml)	-0.06	0.67	-0.12	0.53
IL4 (pg/ml)	-0.16	0.18	0.19	0.23
IL7 (pg/ml)	-0.19	0.12	0.20	0.21
IL8 (pg/ml)	0.03	0.78	0.10	0.50
IP10 (pg/ml)	0.19	0.12	-0.07	0.65
MCP1 (pg/ml)	0.09	0.47	-0.25	0.10
MIP1a (pg/ml)	-0.10	0.43	0.07	0.64
MIP1b (pg/ml)	-0.04	0.72	-0.14	0.37
VEGF (pg/ml)	0.14	0.26	0.07	0.64
CRP (ug/dl)	0.13	0.28	-0.14	0.37
IFNy (pg/ml)	-0.05	0.67	0.11	0.43
IL10 (pg/ml)	0.01	0.95	0.22	0.11
IL13 (pg/ml)	0.14	0.26	0.03	0.86
IL17a (pg/ml)	-0.18	0.13	-0.12	0.40
IL23 (pg/ml)	-0.07	0.55	0.09	0.53
IL5 (pg/ml)	0.00	1.00	0.07	0.64

IL6 (pg/ml)	0.04	0.76	-0.05	0.75
TNFα (pg/ml)	0.00	1.00	0.03	0.84

Note: Data presented as Spearman correlation coefficients.

A

B

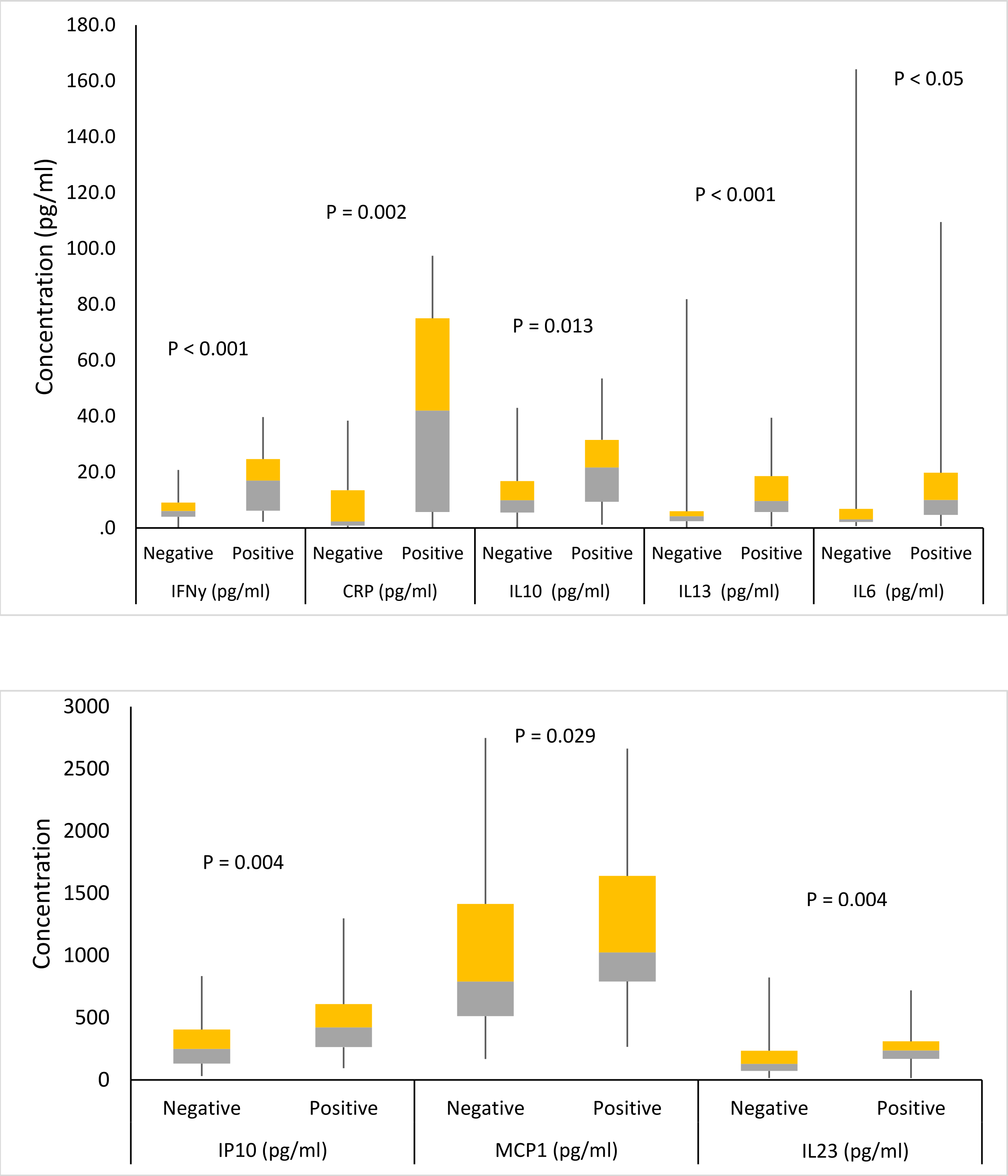

Figure S1. Serum cytokine and chemokine levels of SARS-CoV-2 patients (positive) and healthy controls (negative). Data are expressed as median with 25th and 75th percentiles. Abbreviations: A: IFNy, interferon gamma; CRP, C-reactive protein; IL, interleukin. B: IP-10, IFN-γ inducible protein-10; MCP, monocyte chemoattractant protein, IL, interleukin.
